# Bacteria colonization and gene expression related to immune function in colon mucosa is associated with growth in neonatal calves regardless of live yeast supplementation

**DOI:** 10.1186/s40104-024-01030-7

**Published:** 2024-06-05

**Authors:** Koki Nishihara, Clothilde Villot, Lautaro Cangiano, Le Luo Guan, Michael Steele

**Affiliations:** 1 Department of Animal Biosciences, Animal Science and Nutrition, University of Guelph, Guelph, ON N1G 1Y2 Canada; 2 Department of Agricultural, Food and Nutritional Science, University of Alberta, Edmonton, T6G 2P5 Canada; 3 Lallemand SAS, Blagnac, F-31702 France; 4 Department of Animal and Dairy Sciences, University of Wisconsin-Madison, Madison, WI USA; 5 Faculty of Land and Food Systems, The University of British Columbia, Vancouver, BC V6T 1Z4 Canada; 6 Present Address: Department of Animal Biosciences, University of Guelph, Guelph, ON N1G 2W1 Canada

**Keywords:** Colon biopsy, Dairy calves, RNA-sequencing, *Saccharomyces cerevisiae boulardii*, 16S rRNA amplicon-sequencing

## Abstract

**Background:**

As Holstein calves are susceptible to gastrointestinal disorders during the first week of life, understanding how intestinal immune function develops in neonatal calves is important to promote better intestinal health. Feeding probiotics in early life may contribute to host intestinal health by facilitating beneficial bacteria colonization and developing intestinal immune function. The objective of this study was to characterize the impact of early life yeast supplementation and growth on colon mucosa-attached bacteria and host immune function.

**Results:**

Twenty Holstein bull calves received no supplementation (CON) or *Saccharomyces cerevisiae boulardii* (SCB) from birth to 5 d of life. Colon tissue biopsies were taken within 2 h of life (D0) before the first colostrum feeding and 3 h after the morning feeding at d 5 of age (D5) to analyze mucosa-attached bacteria and colon transcriptome. Metagenome sequencing showed that there was no difference in α and β diversity of mucosa-attached bacteria between day and treatment, but bacteria related to diarrhea were more abundant in the colon mucosa on D0 compared to D5. In addition, qPCR indicated that the absolute abundance of *Escherichia coli* (*E. coli*) decreased in the colon mucosa on D5 compared to D0; however, that of *Bifidobacterium*, *Lactobacillus*, and *Faecalibacterium prausnitzii*, which could competitively exclude *E. coli*, increased in the colon mucosa on D5 compared to D0. RNA-sequencing showed that there were no differentially expressed genes between CON and SCB, but suggested that pathways related to viral infection such as “Interferon Signaling” were activated in the colon mucosa of D5 compared to D0.

**Conclusions:**

Growth affected mucosa-attached bacteria and host immune function in the colon mucosa during the first 5 d of life in dairy calves independently of SCB supplementation. During early life, opportunistic pathogens may decrease due to intestinal environmental changes by beneficial bacteria and/or host immune function. Predicted activation of immune function-related pathways may be the result of host immune function development or suggest other antigens in the intestine during early life. Further studies focusing on the other antigens and host immune function in the colon mucosa are required to better understand intestinal immune function development.

**Supplementary Information:**

The online version contains supplementary material available at 10.1186/s40104-024-01030-7.

## Background

Gastrointestinal disorders remain the most common cause of morbidity and mortality for dairy calves in North America [1, 2]. Urie et al. [1] reported that the percentage of morbidity and mortality of heifers during the pre-weaning period were 33.8% and 5.0%, and gastrointestinal disease contributed 43.6% and 32.0%, of all recorded morbidity and mortality events, respectively. The reason why calves are more susceptible during this period may be that their immune system is naive at birth and not yet fully developed [3]. Especially, in the first few days of life, the calf’s immunity depends on the transfer of passive immunity exclusively from colostrum intake [4]. In a study conducted by Urie et al. [1], weekly incidence rates of gastrointestinal disorders peaked at 2 weeks of age, suggesting that the transfer of passive immunity from colostrum is not enough to prevent pathogenic bacteria colonizing the intestine during early life of dairy calves.

Bacterial colonization in the intestine could impact host immune function development in other mammalian species [5]. Comparisons of germ-free and conventionally raised rodents showed that postneonatal microbial colonization and intestinal exposure play crucial roles in promoting mucosa immune development by stimulating mucus production, epithelial cell proliferation, and immunocompetence [6, 7]. The use of gnotobiotic animals revealed that specific beneficial bacterial colonization of the intestine in early life alters host immune function and affects susceptibility to colitis [8]. The usage of antibiotic during early life could impair beneficial bacteria in the intestine [9, 10] and result in subsequent diarrhea [11]. Thus, facilitating beneficial bacteria colonization in the intestine may alter host immune function and prevent gastrointestinal disorders in neonatal calves. Understanding the dynamics of bacteria and transcriptome profiles in the colon mucosa during early life may elucidate why calves are susceptible to gastrointestinal disorders during this period. It may also aid in identifying the key host immune functions and beneficial bacteria in the colon mucosa, thereby preventing gastrointestinal disorders.

One strategy to facilitate beneficial bacterial colonization and prevent gastrointestinal disorders is to feed probiotics [12–14]. *Saccharomyces cerevisiae boulardii* (SCB) is a live yeast, defined as a probiotic. Supplementation of SCB reduced the abundance of potentially opportunistic pathogens in the colon mucosa of pre-weaning calves [15] and increased beneficial bacteria abundance in the jejunum and feces [16, 17]. It also reduced the incidence of severe diarrhea in pre-weaned calves [17]. In addition, it was shown that supplementing SCB to newborn calves from birth stimulates secretory immunoglobulin A (sIgA) production in the intestine at 1 week of age [16]. Taken together, these suggest that SCB supplementation in early life could inhibit opportunistic pathogens colonization, promote beneficial bacteria colonization, and develop the immune system in the intestine of neonatal calves. However, the comprehensive contribution of SCB supplementation to bacterial colonization and host immune function in the colon during early life remains unclear.

The objective of this study was to characterize the ontological-associated changes in microbial colonization and mucosal immune development, as well as the influence of SCB supplementation on these processes. Some studies have reported the relationship between growth and intestinal bacterial colonization or transcriptomes in the colon mucosa during early life [18–23]; however, these studies compared different ages and used different animals. To evaluate the effect of growth, we collected colon biopsy samples from the same individuals at different time points. We hypothesized that early yeast supplementation prevents the colonization of opportunistic pathogens, promotes colonization of beneficial bacteria, and develops host immune function in the colon mucosa, and that growth is associated with opportunistic pathogens colonization and higher expression of immune function-related genes in the colon mucosa.

## Methods

### Animal study

Animals and diets were described in our previous study [16]. In brief, this experiment was conducted at the Dairy Research and Technology Center of the University of Alberta under the approval of the University of Alberta Animal Care and Use Committee for Livestock (AUP00002645). Holstein heifers and cows were moved to maternity pens 3 to 10 d before expected parturition. A clean and disinfected birth-monitoring sensor (iVET^®^, Papenburg, Germany) [24] was inserted into the vagina in order to accurately detect the calving process of each cow. A total of 20 Holstein bull calves born between May and August 2018, with birth body weight (BW) between 35 and 55 kg, were used in this study and randomly assigned to a unique pen referring to 1 of the 2 dietary treatments for 5 d. Naturally delivered singlet calves were removed from their dams immediately after birth to avoid any contact. Birth BW was recorded before transferring the calf to an individual and disinfected indoor pen (150 cm × 125 cm) bedded with a consistent amount of fresh straw. Newborns were randomly assigned based on their date of birth and initial body weight to a specific pen, representing 1 of the 2 dietary treatments for 1 week. All calves were then dried with clean towels for 10 min and naval-dipped using 7% iodine before starting any procedures. The animals included in this study presented healthy conditions at birth based on general appearance, rectal temperature, umbilical aspect, and nasal discharge.

### Feeding

Calves received 2 meals of standardized colostrum (HeadStart, Saskatoon Colostrum Co. Ltd., Saskatoon, SK, Canada) at 2 h [7% of birth BW, 180 g of immunogloblin G (IgG) fed] and 12 h (3% of birth BW, 120 g of IgG fed). Colostrum powder was hydrated with warm water (42 °C) to make the final volume required for each calf and fed initially by a bottle. The remainder of the meal volume was tube-fed if the calves did not consume all the meal within 30 min. Calves were subsequently bottle-fed daily 2 meals of milk replacer (MR) at 0700 and 1900. The MR used in this study contained 260 g/kg of crude protein, 160 g/kg of crude fat, and 4.58 Mcal/kg of metabolizable energy on a dry matter basis (Grober Animal Nutrition, Cambridge, Ontario, Canada), and 150 g of powder was mixed with water (42 °C) to make 1 L of MR solution. The initial volume of the individual meal offered to each calf was 7.5% of their birth BW during the 2 first days of the experiment and increased to 8.5% of d 2 BW from d 3 to 5. The refusal of the milk replacer was recorded individually for each feeding. Calves were given ad libitum access to water. Ten calves (CON) did not receive supplementation in any of their meals during the entire experiment. The other 10 calves (SCB) were supplemented throughout the experiment with the same daily quantity of 5 g of live SCB (ProTernative Milk; containing 2 × 10^9^ CFU/g of live *Saccharomyces cerevisiae boulardii* CNCM I-1079 strain; Lallemand Animal Nutrition, Montreal, Quebec, Canada). Supplementation was added to the first colostrum feeding and subsequently to each MR morning feeding, which was expected to supply 10 × 10^9^ CFU of SCB per calf per day. In order to ensure the total consumption of the supplementation, a pre-morning meal was prepared by mixing 5 g of live SCB with 260 mL (part of an allocated meal) of either colostrum or MR solution, and was fed to the SCB calves before they received the remainder of their meal.

### Colon biopsies sampling

Colon tissue biopsies were taken within 2 h of life (D0) and 3 h after morning feeding on d 5 of age (D5), according to the methodology developed by van Niekerk et al. [25], which ensured suitable samples for colon mucosa-attached bacteria and gene expression analysis. Briefly, the calves were laid down and gently restrained during sampling. The lubricated distal tip of the endoscope was gradually inserted into the calf’s anus. Colon tissue samples were collected per calf per time point from the distal colon (30–40 cm from the calf’s anus) with endoscopic biopsy forceps (Captura hot biopsy forceps, HDBF-2.4-230-S, Cook Medical). The tissue samples were immediately washed in sterilized phosphate-buffered saline (10 mmol/L sodium phosphate, pH 7.2, containing 130 mmol/L sodium chloride) in order to remove the non-adhered bacteria. Samples were stored in RNA stabilization fluid (RNALater, ThermoFisher Scientific, Burlington, ON, Canada) at room temperature for 24 h and then frozen at −80 °C for later DNA and RNA extraction.

### DNA extraction and amplicon sequencing

Total genomic DNA was extracted from colon biopsies using Qiagen DNeasy Blood and Tissue kit (Qiagen, Valencia, CA) according to the manufacturer’s instructions. The quality and quantity of the DNA were assessed using a Nanodrop ND-1000 spectrophotometer (NanoDrop Technology, Wilmington, DE, USA). To explore the colon mucosa-attached bacterial profile, the bacterial hypervariable V1–V3 regions of the 16S rRNA gene were amplified using the primers {9F (5´-GAGTTTGATCMTGGCTCAG-3´) and 515R (5´-CCGCGGCKGCTGGCAC-3´) [26]}. Amplicons were sequenced using Illumina MiSeq PE300 to generate paired-end sequences.

### Bioinformatic analysis of colon mucosa-attached bacteria

The raw sequences were processed using the available plugins in QIIME2 v. 2019.4 [27]. First, fastq files, primer and adapter sequences trimmed, error correction and paired-end merging and identification of amplicon sequence variants (ASV) were conducted with DADA2 [28]. Subsequently, the representative sequences were aligned with MAFFT [29], followed by the creation of a phylogenetic tree using FastTree2 [30]. Taxonomy assigned to ASVs with Naïve-Bayes classifier trained using the V1–V3 regions of 16S sequences from Silva database (v128) [31]. Two samples (C12-D5 and C20-D5) with a Feature Count lower than 3,000 were filtered, and the other samples had a Feature Count higher than 8,000. Bacteria present in at least 1 sample and with a relative abundance > 0.01% were defined as detectable and were used for further analysis. The tree and feature table were used to analyze α and β diversity using core diversity metrics plugin in QIIME2, including Observed OTUs, Shannon, and Chao1 indices for α diversity and weighted and unweighted UniFrac distances for β diversity.

### Quantification of specific microbial groups in colon biopsy

After DNA extraction, DNA was further purified using QIAmp fast DNA stool mini kit (Qiagen Inc., Germantown, MD, USA). The quantity and purity of DNA were evaluated using a NanoDrop 1000 spectrophotometer, and DNA was stored at −20 °C until further use. The abundance of total bacteria, *Bifidobacterium*, *Lactobacillus*, *Faecalibacterium prausnitzii*, and *Escherichia coli* was estimated by measuring their respective 16S rRNA gene copy numbers using qPCR. The qPCR was performed using SYBR green (Fast SYBR Green Master Mix, Applied Biosystems) with specific primers (Additional file 1: Table S1 [16]) in the high throughput ViiATM 7 Real-Time PCR System (ThermoFisher Scientific, Waltham, MA, USA). Standard curves were generated for each bacterial group using purified plasmids carrying 16S rRNA genes of *Bifidobacterium adolescentis*, *Lactobacillus acidophilus* ATCC4356, *Faecalibacterium prausnitzii* (*F. prausnitzii*) A2–165, and *E. coli* K12. The copy number of the 16S rRNA gene per gram of fresh matter was calculated using following equation [32]:

$$(QM \times C \times DV)/(S \times W),$$

where *QM* was the quantitative mean of copy number obtained from qPCR; *C* was the DNA concentration of each sample (ng/µL); *DV* was the dilution volume of extracted DNA (µL); *S* was the DNA amount subjected to the qRT-PCR analysis (ng); and *W* was the sample weight subjected to the DNA extraction (g).

### Statistical analysis

Differential abundance testing for the relative abundance of bacteria detected using amplicon-sequencing and the absolute abundance of total and specific bacteria (*Bifidobacterium*, *Lactobacillus*, *F. prausnitzii*, and *E. coli*) between D0 vs. D5 was performed by paired Wilcoxon signed-rank test using stats packages in R software (v4.1.3). Two samples (C12-D0 and C20-D0) which paired with 2 missing samples (C12-D5 and C20-D5) were excluded from differential abundance testing for amplicon-sequencing using paired Wilcoxon signed-rank test. Differential abundance testing of total and specific bacteria between CON vs. SCB on D0 or D5 and between D0 vs. D5 in CON or SCB was performed by Wilcoxon signed-rank test using stats packages in R software. The *P*-values (both for amplicon-sequencing and qPCR data) were adjusted with Benjamini–Hochberg approach for false discovery rate (FDR) with statistical significances declared at FDR < 0.05.

### RNA extraction and sequencing

Biopsied colon tissues were ground (~0.1 g) in liquid nitrogen before RNA extraction. Samples were then homogenized with TRIzol (Invitrogen, Grand Island, NY) and CK-14 Precellys lysine kit (Bertin Technologies, Montigny, France) using a Precellys homogenizer. Then, the lysate was treated with chloroform, isopropanol, and high salt solution (1.2 mmol/L NaAc, 0.8 mol/L NaCl) sequentially. The RNA was precipitated using cold ethanol and dissolved in nuclease-free water (200 µL). The RNA concentration was measured using NanoDrop 1000 spectrophotometer and Qubit 3.0 Fluorometer (Invitrogen, Carlsbad, CA, USA) and the RNA integrity number [mean = 7.07 (± 0.77 SD), max = 8.4, min = 5.3] was measured using Agilent 2200 Tape station (Agilent Technologies, Santa Clara, CA, USA). Library construction was performed using the TruSeq RNA Library Prep kit v2 (Illumina, San Diego, CA, USA). Adapters were ligated to the ends of double-stranded cDNA and PCR amplified to create libraries. Thereafter, cDNA libraries were pooled and then sequenced at Génome Québec Innovation Centre (Montréal, Quebec, Canada) using the Illumina HiSeq 4000 PE100.

### mRNA mapping, normalization, principal component analysis, and gene names annotation

Quality control analysis was verified using FastQC (v0.11.4) [33]. The BBDuk package (v38.44) [34] was used for trimming adapters and filtering low-quality bases from the obtained sequences by keeping length ≥ 50 bp and quality score ≥ 20. Then, the clean sequencing reads were aligned to the bovine reference genome (ARS-UCD1.2) and assembled using STAR package (v2.6.1) [35]. The number of mapped reads per gene was quantified using Subread package (v1.6.1) [36]. The mapped reads were normalized using counts per million (CPM) as follows: (number of reads mapped to a gene in a sample)/(total number of reads mapped to all annotated genes in a sample) × 10^6^. Genes were considered expressed when CPM > 1 in at least 5 animals in at least 1 age group. A principal component analysis (PCA) was performed using ggfortify (v0.4.14) package in R software. Associated gene name annotation was performed using biomaRt (v2.50.3) package in R software [37].

### Bioinformatic analysis of colon transcriptome analysis

Bioconductor edgeR (v3.36.0) package [38] in R software was used to identify differential expressed (DE) genes between D0 vs. D5 and between CON vs. SCB. All calves (*n* = 20) and days (D0 and D5) were included in the GLM model. Initially, treatment (CON and SCB) was included in the GLM model, but it was later excluded from the model because the coefficients of treatment were not estimable. Genes with |log_2_ fold change (logFC)| > 1.5 and FDR < 0.05 were considered DE and selected for further functional analysis. Database for Annotation, Visualization, and Integrated Discovery (DAVID) (v2022q3) [39] was used for Gene Ontology (GO) terms and Kyoto Encyclopedia of Genes and Genomes (KEGG) enrichment of commonly expressed genes among groups and uniquely expressed genes in each time-point and treatment. Metabolic pathway analysis of DE genes was performed using Ingenuity Pathway Analysis (IPA; QIAGEN, Redwood City, www.qiagen.com/ingenuity). Ingenuity canonical pathways (FDR < 0.05, and |z-score| > 2) were sorted by FDR in ascending order, and the top 5 were considered up- and down-regulated pathways.

### Weighted correlation network analysis

To examine the relationship between expressed genes in the colon mucosa at D0 and D5 and bacteria in the colon mucosa (total bacteria, *Faecalibacterium prausnitzii*, and *Escherichia coli*, *Bifidobacterium*, and *Lactobacillus*), the weighted correlation network analysis (WGCNA) package (v1.72.1) [40] in R software was used to identify the co-expressed genes correlated with the traits. Genes considered expressed were normalized by log_2_ transformed [log_2_(CPM + 1)] and inputted to WGCNA. Sample clustering based on expressed genes showed no outliers in the samples (Additional file 2: Fig. S1A); therefore, no samples were removed in WGCNA. When “RsquaredCut” was set at 0.85 and “networkType” was assigned as signed using “pickSoftThreshold” function, the minimum power value was 18 (Additional file 2: Fig. S1B). The power value was set to 14 because the connectivity distribution was decrescent and the network was considered following the scale-free topology criterion (Additional file 2: Fig. S1C–E). The co-expressed genes were grouped into modules (distinguished by different color names) using the automatic 1-step function, “blockwiseModules” with the following parameters: “networkType” = signed and “minModuleSize” = 60. The other parameters in “pickSoftThreshold” and “blockwiseModules” functions were followed by default. The modules were then used to identify module–trait relationships (calculated by Spearman correlation) with bacteria in the colon mucosa. The gene modules with *P* < 0.05 were considered correlated to a trait. The thresholds used for correlation (*r*) were 0 to 0.29 as weak, 0.3 to 0.69 as moderate, and 0.7 to 1.0 as strong for the absolute values for *r*.

## Results

### Overview of 16S rRNA amplicon-sequencing

**Fig. 1:**
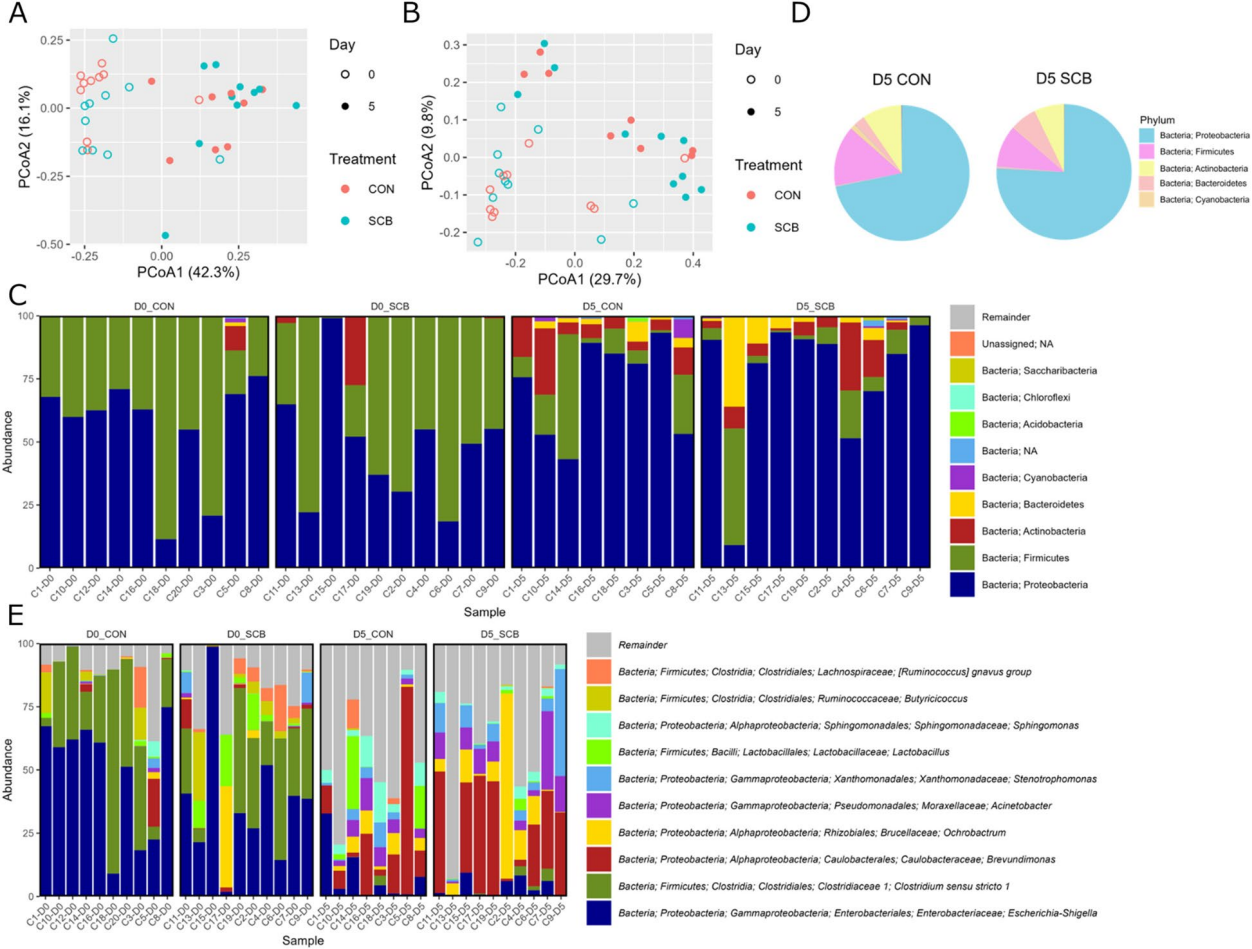
Overview of colon mucosa-attached bacteria. Principal coordinate analysis (PCoA) presents the bacterial community in the colon mucosa based on β-diversity [(**A**) weighted and (**B**) unweighted UniFrac distances]. The PCoA was plotted using qiime2R. **C **Top 10 bacterial profiles at the phylum level in each calf. **D **Relative abundance of taxonomy (phylum level) on D5 CON and SCB. **E** Top 10 bacterial profiles at the genus level in each calf. PCoA: Principal coordinate analysis; SCB: *Saccharomyces cerevisiae boulardii*

A total of 2,214,640 sequences were generated with an average of 58,280 (± 17,853 SD) sequences/sample. There were no significant differences in Chao1 [D0 = 74.6 (± 6.1 SE), D5 = 75.3 (± 11.4 SE), *P* > 0.05] and Shannon index [D0 = 4.6 (± 0.2 SE), D5 = 4.3 (± 0.3 SE), *P* > 0.05] of colon mucosa-attached bacteria between D0 and D5. Also, there were no significant differences in Chao1 [D5 CON = 84.4 (± 23.9 SE), D5 SCB = 68.1 (± 8.9 SE), *P* > 0.05] and Shannon [D5 CON = 4.6 (± 0.4 SE), D5 SCB = 4.2 (± 0.3 SE), *P* > 0.05] between D5 CON and D5 SCB. Regarding β diversity, principal coordinate analysis (PCoA) and permutational analysis of variance based on weighted and unweighted UniFrac distances matrix showed that colon mucosa-attached communities clustered together according to the sampling day (weighted UniFrac distance: pseudo-F = 17.0; *P* = 0.001; unweighted UniFrac distance: pseudo-F = 6.1; *P* = 0.001; Fig. 1A and B). There were no significant differences between CON and SCB in D5 β diversity (weighted UniFrac distance: pseudo-F = 0.6; *P* > 0.05; unweighted UniFrac distance: pseudo-F = 0.9; *P* > 0.05). Taxonomic analysis identified a total of 15 detectable bacterial phyla in the colon mucosa (Fig. 1C and Additional file 1: Table S2). Among them, Proteobacteria [52% (D0) and 74% (D5)], Firmicutes [46% (D0) and 12% (D5)], and Actinobacteria [2% (D0) and 8% (D5)] were the top 3 abundant bacterial phyla on both day (Table 1). In addition, at D5, the top 3 most abundant bacterial phyla were Proteobacteria [72% (CON) and 76% (SCB)], Firmicutes [14% (CON) and 10% (SCB)], and Actinobacteria [9% (CON) and 7% (SCB)] in both the CON and SCB groups (Fig. 1D). A total of 308 bacteria were identified at the genus level in the colon mucosa (Fig. 1E and Additional file 1: Table S3). On D0, the top 3 abundant bacterial genera were *Escherichia-Shigella* (42%), *Clostridium sensu stricto 1* (28%), and *Butyricicoccus* (4%) in D0, whereas *Brevundimonas* (24%), *Ochrobactrum* (9%), and *Acinetobacter* (7%) on D5 (Table 1).

**Table 1:** Top 10 abundant bacteria at phylum and genus level

**D0**		**D5**	
**Phylum**			
Proteobacteria	51.99%	Proteobacteria	73.88%
Firmicutes	45.63%	Firmicutes	11.94%
Actinobacteria	2.07%	Actinobacteria	8.20%
Cyanobacteria	0.14%	Bacteroidetes	4.61%
Bacteroidetes	0.11%	Cyanobacteria	0.66%
Chloroflexi	0.01%	Unassigned	0.46%
Unassigned	0.06%	Acidobacteria	0.08%
		Saccharibacteria	0.06%
		Chloroflexi	0.05%
		Spirochaetae	0.02%
**Genus**			
*Escherichia-Shigella*	41.92%	*Brevundimonas*	24.27%
*Clostridium sensu stricto 1*	28.28%	*Ochrobactrum*	8.84%
*Butyricicoccus*	3.89%	*Acinetobacter*	7.15%
*[Ruminococcus] gnavus group*	3.27%	*Stenotrophomonas*	5.75%
*Lactobacillus*	2.78%	*Escherichia-Shigella*	5.44%
*Enterococcus*	2.67%	*Sphingomonas*	4.13%
*Ochrobactrum*	2.32%	*Lactobacillus*	3.03%
*Brevundimonas*	2.14%	*Rhizobium*	2.62%
*Propionibacterium*	1.48%	*Bradyrhizobium*	2.31%
*Tyzzerella 4*	1.40%	*Pseudomonas*	2.30%

### Differential abundance testing in colon mucosa-attached bacteria

**Fig. 2:**
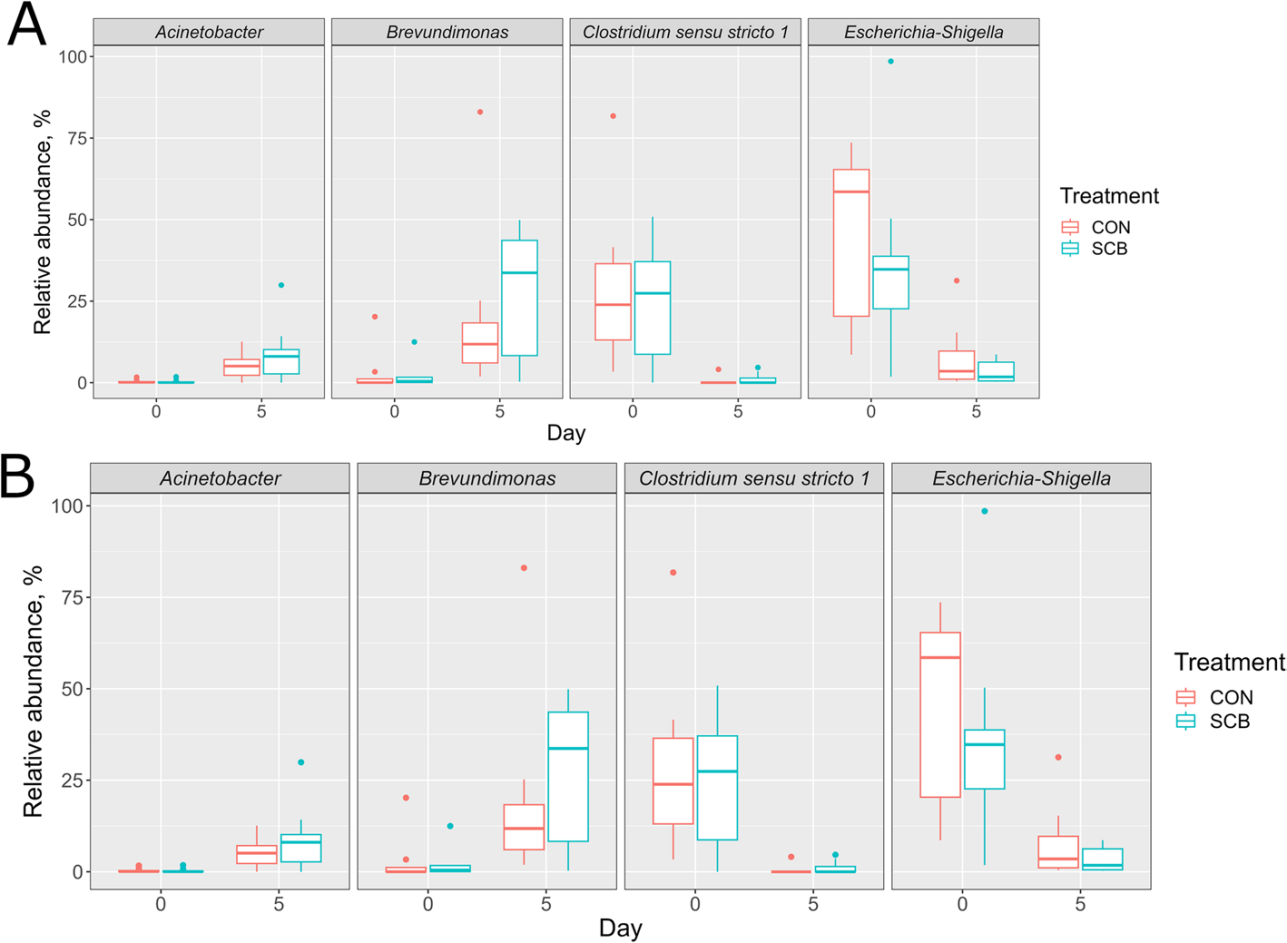
Differentially abundant bacteria (FDR < 0.05) in the colon mucosa between D0 and D5. Differentially abundant bacteria at the phylum level (**A**) and at the genus level (**B**) were detected using Wilcoxon signed-rank test. The boxes show the interquartile range between the first and third quartiles and the line inside the box defines the median. Individual data points beyond the whiskers are depicted as dots. FDR: false discovery rate

Differential abundance testing using Wilcoxon signed-rank test revealed that at the phylum level, Actinobacteria, Bacteroidetes, and Proteobacteria were more abundant in the colon mucosa on D5 than on D0 (FDR < 0.05, respectively), while Firmicutes was more abundant in the colon mucosa on D0 than on D5 (FDR < 0.05) (Fig. 2A). At the genus level, the relative abundances of *Acinetobacter* and *Brevundimonas* were higher on D5 than on D0 (FDR < 0.05, respectively), while *Clostridium sensu stricto 1* and *Escherichia-Shigella* were more abundant on D0 than on D5 (FDR < 0.05, respectively) (Fig. 2B). There were no significant differences in the abundance of colon mucosa-attached bacteria between the CON and SCB groups on D0 or D5. Comparisons between D0 CON vs. D5 CON showed that Actinobacteria and Bacteroidetes were more abundant on D5 CON than on D0 CON, while Firmicutes were more abundant in D0 CON than in D5 CON (FDR < 0.05, respectively). These significant differences were also found between D0 SCB vs. D5 SCB (FDR < 0.05, respectively). At the genus level, *Clostridium sensu stricto 1* and *Escherichia-Shigella* were more abundant on D0 CON than on D5 SCB (FDR < 0.05, respectively), while only *Escherichia-Shigella* was more abundant on D0 SCB than on D5 SCB (FDR < 0.05, respectively).

### Longitudinal bacterial abundance

**Fig. 3:**
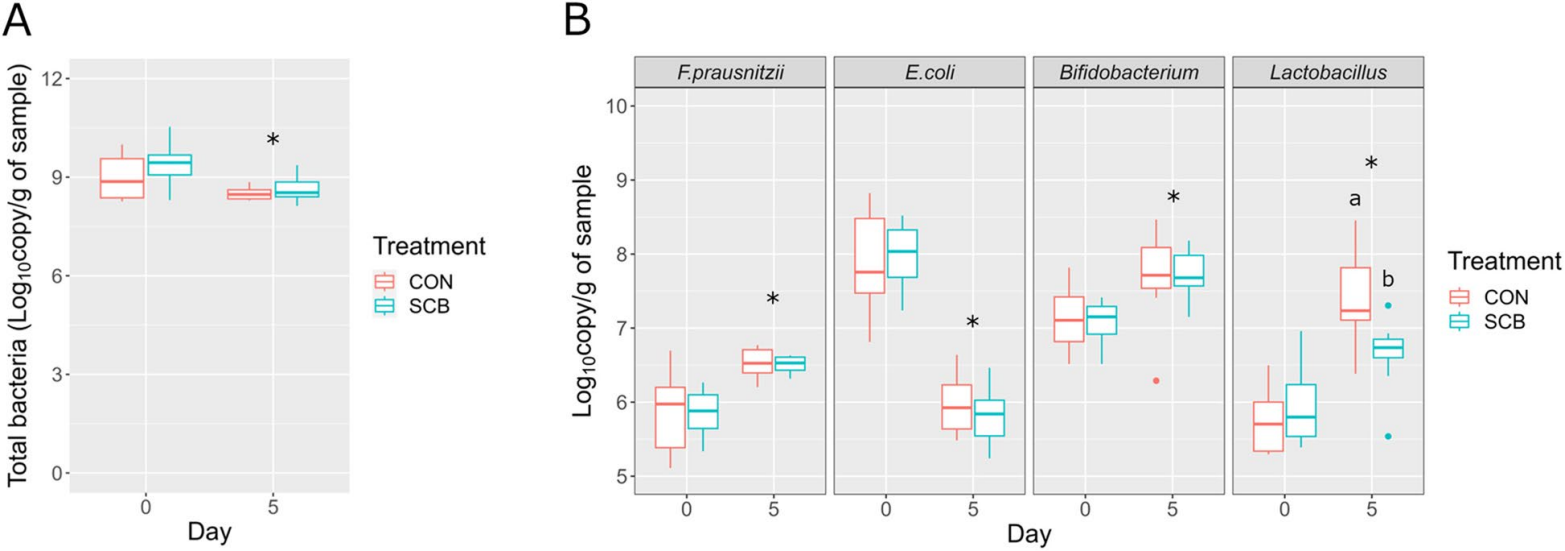
The abundance of total and 4 bacteria groups in the colon mucosa. Density of total bacteria (**A**) and absolute abundance of 4 bacterial groups (**B**) on D0 and D5 in the colon mucosa of CON and SCB calves. The boxes show the interquartile range between the first and third quartiles and the line inside the box defines the median. A significant time effect was found in all comparisons. Individual data points beyond the whiskers are depicted as dots. ^a,b^Different letters above boxplot indicate a significant difference between CON and SCB on D5 (FDR < 0.05), and * above boxplot on D5 indicates a significant difference between D0 and D5 groups (FDR < 0.05) using Wilcoxon signed-rank test. SCB: *Saccharomyces cerevisiae boulardii*; FDR: false discovery rate

The total bacteria and absolute abundance of *F. prausnitzii*, *E. coli*, *Bifidobacterium*, and *Lactobacillus* are shown in Fig. 3. Total bacteria (log_10_ copy/g of sample) decreased on D5 compared to those on D0 (FDR < 0.05) (Fig. 3A). While the absolute abundance of *E. coli* decreased on D5 compared to those on D0 (*P* < 0.05), the absolute abundance of *F. prausnitzii*, *Bifidobacterium*, and *Lactobacillus* increased on D5 compared to those on D0 (FDR < 0.05) (Fig. 3B). Only the absolute abundance of *Lactobacillus* of CON calves was higher than that of SCB (FDR > 0.05).

### Overview of transcriptome

**Fig. 4:**
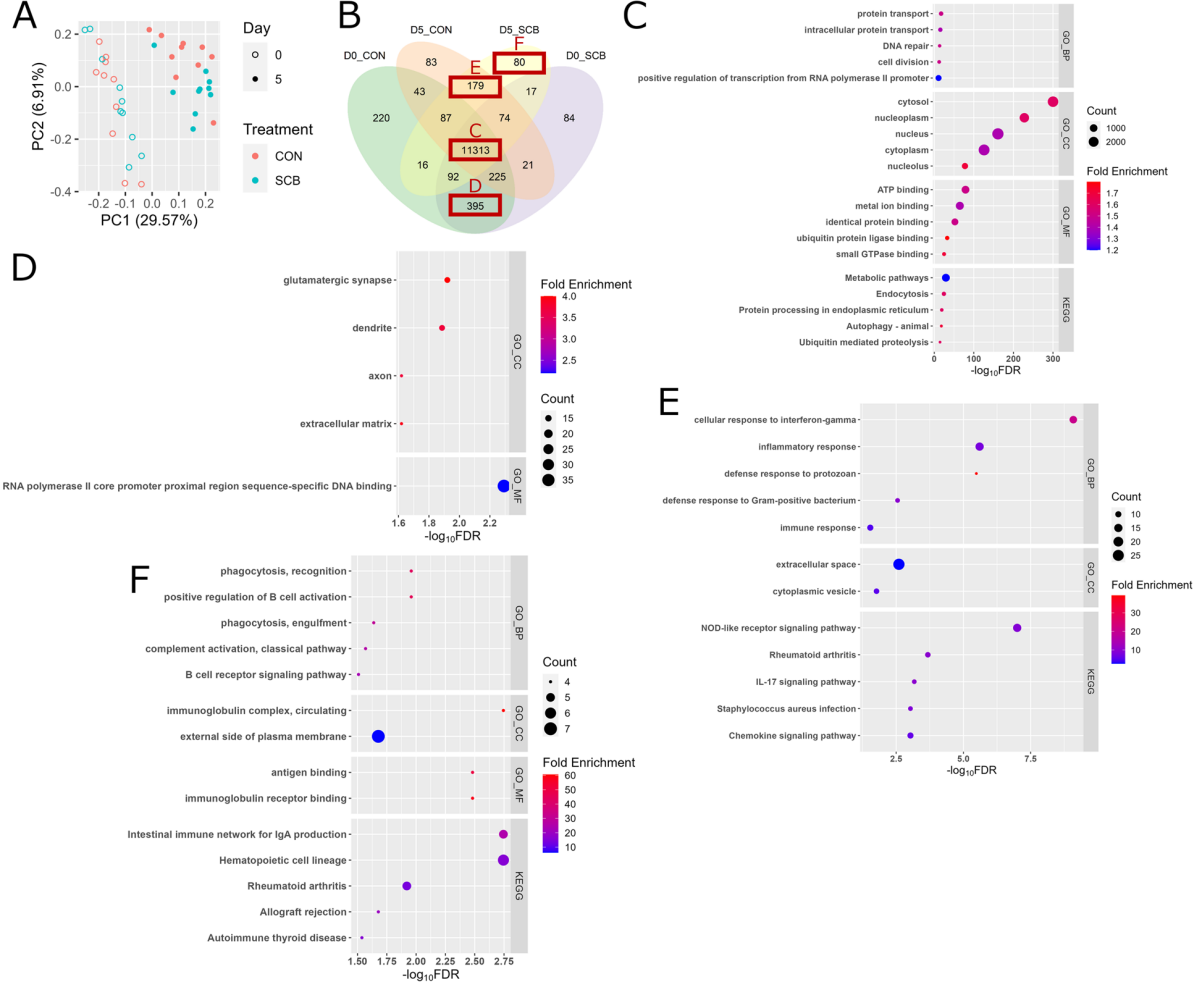
Overview of the transcriptome. **A** PCA plot of expressed the colon mucosa transcriptomes in each calf. **B** Venn diagram of expressed genes in the colon mucosa among groups. Top 5 biological processes, cellular components, molecular function, and KEGG pathways of shared genes among groups (**C**), uniquely expressed genes on D0 and commonly expressed between CON and SCB (**D**), uniquely expressed genes on D5 and commonly expressed between CON and SCB (**E**), and uniquely expressed genes in D5 SCB (**F**) as identified by DAVID. PCA: principal component analysis; KEGG: Kyoto Encyclopedia of Genes and Genomes; DAVID: Database for Annotation, Visualization, and Integrated Discovery; SCB: *Saccharomyces cerevisiae boulardii*

A total of 1,236,882,937 paired-end reads were generated, with an average of 30,922,073 (± 3,826,227 SD) paired-end reads. In the colon tissue of calves, 12,929 genes were totally expressed (CPM > 5 and in more than 5 out of 10 animals per group) (Additional file 1: Table S4). The PCA plot (Fig. 4A) indicated that the transcriptome profiles of D0 and D5 clustered differently, whereas the CON and SCB groups did not exhibit clear clustering. A total of 12,391, 12,221, 12,025, and 11,858 genes were expressed in D0 CON, D0 SCB, D5 CON, and D5 SCB, respectively (Fig. 4B), with 11,313 genes being commonly expressed among them (Additional file 1: Table S5). In addition, 220, 84, 83, and 80 genes were identified as uniquely expressed genes for D0 CON, D0 SCB, D5 CON, and D5 SCB, respectively (Additional file 1: Table S6–9). Annotation using DAVID showed that the top enriched (FDR < 0.05 and fold enrichment > 1) biological processes, cellular components, molecular functions, and pathways of commonly expressed genes were “protein transport”, “cytosol”, “ATP binding” and “Metabolic pathways”, respectively (Fig. 4C). The top enriched cellular components and molecular function were “glutamatergic synapse” and “RNA polymerase II core promoter proximal region sequence-specific DNA binding” in genes uniquely expressed on D0 and commonly expressed between CON and SCB, respectively (Fig. 4D), whereas the top enriched biological processes, cellular components, and pathways were “cellular response to interferon-gamma”, “extracellular space” and “NOD-like receptor signaling pathway” in genes uniquely expressed in D5 and commonly expressed between CON and SCB, respectively (Fig. 4E). In the uniquely expressed genes of D0 CON, “potassium ion transmembrane transport” and “integral component of plasma membrane” were annotated as enriched biological processes and cellular components, respectively. In the uniquely expressed genes of D5 CON, “inflammatory response” was annotated as an enriched biological process. The top enriched (FDR < 0.05 and fold enrichment > 1) biological processes, cellular components, molecular functions, and pathways of uniquely expressed genes in D5 SCB were “positive regulation of B cell activation”, “immunoglobulin complex, circulating”, “immunoglobulin receptor binding” and “Hematopoietic cell lineage”, respectively (Fig. 4F and Additional file 2: Fig. S2). No terms and pathways were enriched in commonly expressed genes between D0 and D5 CON and in those between D0 and D5 SCB.

### DE genes analysis and WGCNA in the colon tissues

**Fig. 5:**
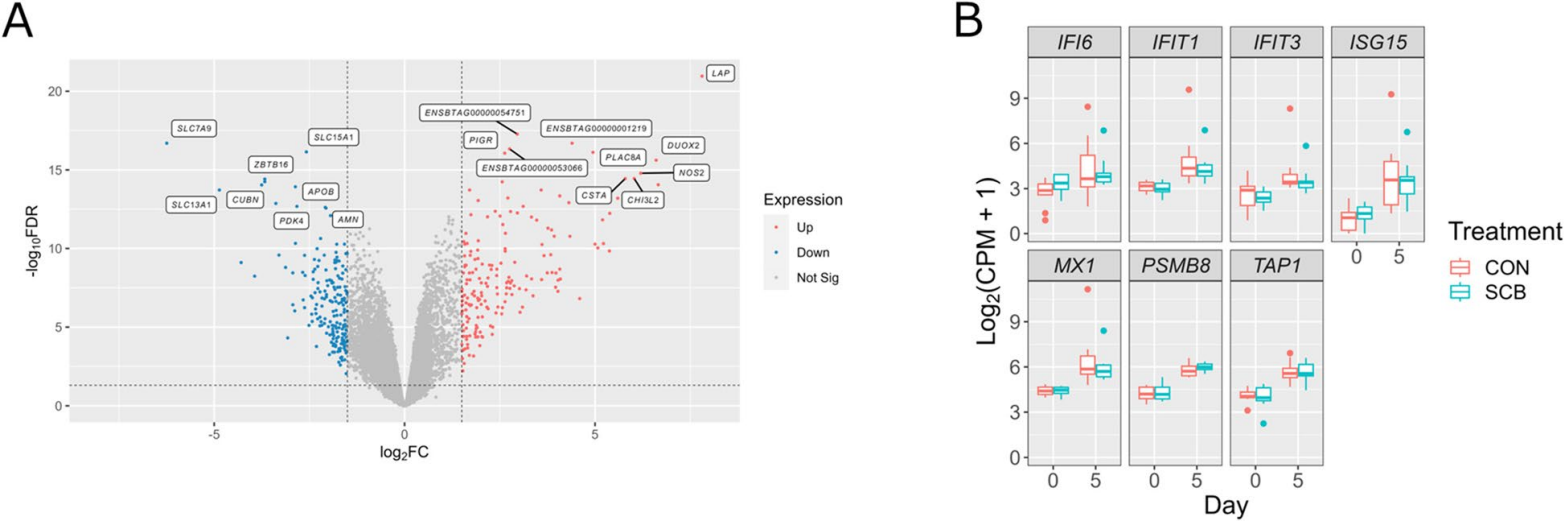
DE genes between D0 vs. D5 in the colon mucosa. **A **Volcano plot of expressed genes and DE genes between D0 and D5. *X*-axis shows log_2_ fold change (log_2_FC) and *y*-axis shows –log_10_ false discovery rate (−log_10_FDR). The threshold of log_2_FC (> |1.5|) and −log_10_FDR (> 1.30) were dash-ploted in grey color. Down-regulated genes were colored by blue, up-regulated genes were colored by red, and non-DE genes were colored by grey. Gene names of top 10 down- and up-regulated genes based on FDR were annotated. **B **log_2_(CPM + 1) of DE genes involved in “Interferon Signaling” in D0 and D5 of CON and SCB calves. The boxes show the interquartile range between the first and third quartiles, and the line inside the box defines the median. Outliers are shown as individual dots. DE: differential expressed; log_2_FC: log_2_ fold change; FDR: false discovery rate; CPM: counts per million

There were 371 DE genes (179 up-regulated and 192 down-regulated) between D0 vs. D5 (Fig. 5A and Additional file 1: Table S10). Functional analysis of DE genes using IPA showed the top 5 enriched metabolic pathways (Table 2). Among them, “Interferon Signaling”, “Pathogen Induced Cytokine Storm Signaling Pathway”, “Macrophage Classical Activation Signaling Pathway” and “Role of Hypercytokinemia/hyperchemokinemia in the Pathogenesis of Influenza” were predicted to be activated in D5 compared to D0, whereas “LXR/RXR Activation” was predicted to be inhibited in D5 compared to D0. Seven DE genes involved in “Interferon Signaling” were interferon alpha inducible protein 6 (*IFI6*), Interferon induced protein with tetratricopeptide repeats 1 (*IFIT1*), Interferon induced protein with tetratricopeptide repeats 3 (*IFIT3*), Interferon-stimulated gene 15 (*ISG15*), MX dynamin like GTPase 1 (*MX1*), Proteasome 20 S subunit beta 8 (*PSMB8*), and Transporter 1, ATP binding cassette subfamily B member (*TAP1*) (Fig. 5B).

**Table 2:** Top 5 metabolic pathways enriched in D5 compared to D0 using IPA

Ingenuity canonical pathways	−log_10_FDR	z-score	Molecules
Interferon Signaling	4.23	2.646	*IFI6, IFIT1, IFIT3, ISG15, MX1, PSMB8, TAP1*
Pathogen Induced Cytokine Storm Signaling Pathway	3.69	2.668	*C3, CCL20, CCL25, CCL8, COL2A1, CSF2RB, CXCL2, CXCL6, CXCL8, GSDME, HLA-DMB, IFIH1, IL1B, IL1RN, NLRC5, NOS2, ZBP1*
Macrophage Classical Activation Signaling Pathway	3.69	2.887	*ACOD1, CCL20, CD40, CXCL8, GBP4, HLA-DMB, IL1B, LBP, MAF, NOS2, PARP14, PARP9*
Role of Hypercytokinemia/hyperchemokinemia in the Pathogenesis of Influenza	3.37	2.828	*CXCL8, IFIT3, IL1B, IL1RN, ISG15, ISG20, MX1, RSAD2*
LXR/RXR Activation	2.58	–2.121	*AGT, APOA1, APOB, C3, IL1B, IL1RN, LBP, NOS2*

*IPA *Ingenuity Pathway Analysis, *FDR *False discovery rate, *IFI6 *Interferon alpha inducible protein 6, *IFIT1 *Interferon induced protein with tetratricopeptide repeats 1, *IFIT3 *Interferon induced protein with tetratricopeptide repeats 3, *ISG15 *Interferon-stimulated gene 15, *MX1 *MX dynamin like GTPase 1, *PSMB8 *Proteasome 20 S subunit beta 8, *TAP1 *Transporter 1, ATP binding cassette subfamily B member, *C3 *Complement C3, *CCL20 *C-C motif chemokine ligand 20, *CCL25 *C-C motif chemokine ligand 25, *CCL8 *C-C motif chemokine ligand 8, *COL2A1 *Collagen type II alpha 1 chain, *CSF2RB *Colony stimulating factor 2 receptor subunit beta, *CXCL2 *Chemokine (C-X-C motif) ligand 2, *CXCL6 *Chemokine (C-X-C motif) ligand 6, *CXCL8 *Chemokine (C-X-C motif) ligand 8, *GSDME *Gasdermin E, *HLA-DMB *Major histocompatibility complex, class II, DM beta, *IFIH1 *Interferon induced with helicase C domain 1, *IL1B *Interleukin 1 beta, *IL1RN *Interleukin 1 receptor antagonist, *NLRC5 *NLR family CARD domain containing 5, *NOS2 *Nitric oxide synthase 2, *ZBP1 *Z-DNA binding protein 1, *ACOD1 *Aconitate decarboxylase 1, *CD40 *CD40 molecule, *GBP4 *Guanylate binding protein 4, *LBP *Lipopolysaccharide binding protein, *MAF *MAF bZIP transcription factor, *PARP14 *Poly(ADP-ribose) polymerase family member 14, *PARP9 *poly(ADP-ribose) polymerase family member 9, *ISG20 *Interferon-stimulated gene 20, *RSAD2 *Radical S-adenosyl methionine domain containing 2, *LXR/RXR *Liver X Receptor-Retinoid X Receptor, *AGT *Angiotensinogen, *APOA1 *Apolipoprotein A1, *APOB *Apolipoprotein B

**Fig. 6:**
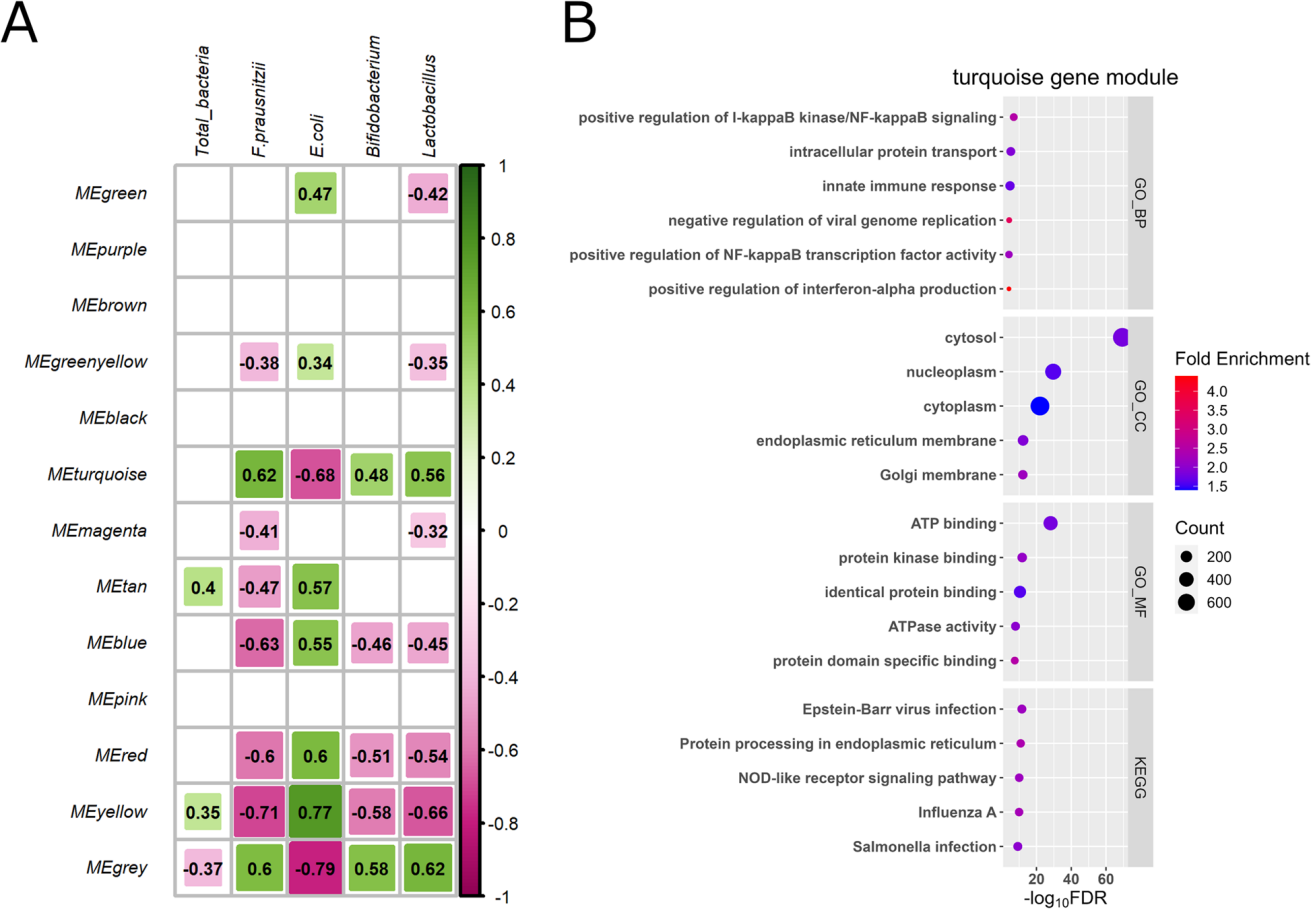
WGCNA identified key colon mucosa modules. **A** WGCNA identification of colon mucosa gene modules correlated with bacteria in the colon mucosa (total bacteria, *F. prausnitzii*, *E. coli*, *Bifidobacterium*, and *Lactobacillus*). Each module name is shown on the left, each trait is shown on the top, and the strength of the correlation is shown as a bar on the right. The correlation coefficients (−1 < *r* < 1) for each module and trait are shown in bold only when the *P*-value was less than 0.05. The turquoise gene modules, which include genes related to immune function, was correlated with bacteria in the colon mucosa. Enriched cellular components, molecular functions, biological functions, and pathways in the turquoise gene module (**B**), as identified by DAVID. WGCNA: weighted correlation network analysis; *F. prausnitzii*: *Faecalibacterium prausnitzii*; *E. coli*: *Escherichia coli*; DAVID: Database for Annotation, Visualization, and Integrated Discovery

A total of 13 gene modules were identified by WGCNA (Fig. 6A). Gene modules (yellow, gray, turquoise, blue, and red), which moderately (|*r*| > 0.3) or strongly (|*r*| > 0.7) correlated with 4 of the bacteria’s absolute abundance (*P* < 0.05), were selected for further functional analysis (Additional file 1: Table S11, Fig. 6B, and Additional file 2: Fig. S3A–C). Functional analysis using DAVID showed that terms and pathways related to immune function were enriched in a turquoise gene module including 3,308 co-expressed genes.

## Discussion

Mucosa-attached bacteria and host immune functions in the colon mucosa of dairy calves in early life have been previously reported [18–21]. However, these studies used different animals and did not wholly explain how growth influences mucosa-attached bacteria and host immune function in early life. We previously developed a methodology for colon biopsy in calves [25], enabling us to elucidate the dynamics of mucosa-attached bacteria and host immune function within the same individuals. Because colon biopsies may yield varying amounts of tissue, access different sampling areas, and entail different contamination risks compared to post-mortem tissue retrieval, the results obtained from colon biopsies may differ from post-mortem sampling. However, this study could offer valuable insights into the ontological development of mucosa-attached bacteria and host immune functions in early life.

Calves in this study were immediately removed from their dams to avoid contact between dams and calves, as in our previous study [20], and colon mucosa samples were collected before the first colostrum feeding. Interestingly, even though we excluded the effects of first colostrum feeding, the relative abundance of colon mucosa in this study demonstrated a result similar to that of our previous study, which collected colon mucosa after colostrum feeding. In this study, 2 phyla (Proteobacteria and Firmicutes) and 2 genera (*Escherichia-Shigella* and *Clostridium sensu stricto 1*) were similarly enriched in the colon mucosa of D0 calves as those fed colostrum [18, 20]. These bacteria may colonize the colon mucosa before or during delivery. Proteobacteria and Firmicutes are detectable in the dams’ amniotic fluid and placenta at early and late gestation, and in the fetal intestine, fetal cecal fluid and tissue, and fetal meconium at gestation [41–43]. *Escherichia-Shigella* and *Clostridium sensu stricto 1* are also detectable in the dam’s amniotic fluid, fetal cecum, and meconium [41, 43]. Thus, these bacteria (or DNA fragments) detected in this study may be derived from the amniotic fluid, placenta, or vagina of the dam. On the other hand, a ruminant species model study, using the fetal lamb [44], supported the hypothesis that the intrauterine and fetal intestine environment of healthy mothers is in a sterile state, similar to studies in humans and mice [45]. Hence, the mucosa-attached microbiota obtained before colostrum feeding may be contaminated with microorganisms originating from reagents used during tissue cleaning, as well as from the skin and vagina of cows, and the farm environment, despite the immediate removal of calves from their dam at birth. Total bacteria of mucosa-attached colon was more abundant before colostrum feeding than after colostrum feeding, which contradicts our previous findings [18, 19]. This, however, could be simply because of contamination. In the future, it is essential to implement contamination controls to exclude these factors from consideration.

*Escherichia-Shigella*, and *Clostridium sensu stricto 1* were more abundant in the feces of diarrheic calves [46, 47], and *Escherichia-Shigella* was more abundant in the colon and ileum of diarrheic piglets [48]. In our study, these 2 genera were more abundant in the colon mucosa on D0 than on D5. Song et al. [19] also reported that the abundance of *Escherichia-Shigella* in the hindgut mucosa were lower pre-weaning compared to birth. In addition, the absolute abundance of *E. coli*, a common opportunistic pathogens in calves intestine [49], decreased during the first 5 d. Thus, relative and absolute abundance of opportunistic pathogens decreased with growth, which is inconsistent with our hypothesis. However, in the study by Song et al. [18], they found that *Escherichia-Shigella* and *Clostridium sensu stricto 1* in the colon mucosa were relatively abundant in 12 h post-neonatal calves compared to 0 and/or 6 h post-neonatal calves, with *Escherichia-Shigella* still the most predominant in D2 colon mucosa [20]. Although we have to consider the differences in the experiments, it is possible that the proportion of these bacteria decreased after at least 12 h of age.

In contrast to *E. coli*, the absolute abundance of *F. prausnitzii*, *Bifidobacterium*, and *Lactobacillus* increased on D5 compared to D0. *Bifidobacterium* and *Lactobacillus* can proliferate under oligosaccharides, glucose, and galactose in colostrum and milk and produce lactic acid [50–53], and *F. prausnitzii* also produces butyric acid in the intestine [54]. Lactic and butyric acid production would result in lower pH in the colon, which may not be optimal pH (6.5–7.5) for *E. coli*, even when *E. coli* can grow over a large range [55]. After birth, anaerobic conditions in the lower gut are maintained by oxygen-consuming bacteria and oxidative chemical reactions [56]. This reduction in oxygen concentration may also be suitable for beneficial bacteria proliferation [57]. In this study, we found a positive correlation between *Bifidobacterium* and *Lactobacillus* with the turquoise gene module, but a negative correlation between *E. coli* with that module (Fig. 6A), which was abundant in genes related to immune response, such as nuclear factor-kappa B (*NF-κB*). This may suggest that host’s immune system interferes with colon mucosa bacterial composition during the first 5 d of life. Indeed, gene expression of antimicrobial peptides [e.g., lingual antimicrobial peptide (*LAP*), dual oxidase 2 (*DUOX2*), and nitric oxide synthase 2 (*NOS2*) [58–60] (Fig. 5A)] were up-regulated at D5. Some antimicrobial peptides could selectively kill pathogenic bacteria [61, 62]. Furthermore, oxidative and nitrosative defenses by *DUOX2* and *NOS2* are involved in anti-bacterial activity of Interferons (IFNs) in phagocytic cells [63]. Considering that “Interferon Signaling” and “Macrophage Classical Activation Signaling Pathway” were ranked among the top 5 metabolic pathways enriched in D5 compared to D0, antimicrobial peptides produced after birth may also contribute to microbiota dynamics during the first 5 d of life in the colon mucosa via the activity of IFN signaling and phagocytic cells. In addition, as polymeric immunoglobulin receptor (*PIGR*), which mediates secretion of immunoglobulin A (IgA) [64], was up-regulated at D5 (Fig. 5A), sIgA secretion in the colon may increase after birth. This may also create an unsuitable environment for opportunistic pathogens by selective exclusion [65]. Thus, from D0 to D5, multiple factors could create a suitable environment for beneficial bacteria while creating an unsuitable environment for opportunistic pathogens. On the other hand, as we conducted PCR specifically for 1 opportunistic bacteria (*E. coli*), dynamics of other opportunistic bacteria remain unclear. Additionally, this study solely focuses on bacteria and excludes other microbial entities like viruses and parasites. Another limitation is the absence of severe diarrhea cases among the calves during the initial 5 d of life in this study. To gain a deeper understanding of diarrhea occurrence in preweaning calves, further research is required to reveal the dynamics of these pathogenic and beneficial bacteria as well as other microbes in the colon mucosa of healthy and diarrheic calves from birth to preweaning.

Although opportunistic pathogens were decreased in the colon mucosa, functional analysis indicates that genes related to immune function were up-regulated at D5. In our previous study, we observed a small number of DE genes in the colon mucosa between the first 0 and 12 h of life [21], suggesting that genes related to immune function in the colon mucosa may be regulated after 12 h of life in the colon mucosa. In this study, 4 immune function-related pathways were predicted to be activated at D5. These may contribute to the reduction of opportunistic pathogens or suggest immune function development associated with growth or antigens from diet or the other pathogens in the colon mucosa during the first 5 d of life. Functional analysis suggests that macrophages may play an important role during the first 5 d of a calf’s life. It is known that macrophage have several faces [66]. Although alternative (M2) macrophages are involved in wound healing and tissue repair, classical (M1) macrophages, which are predicted to be activated in the D5 colon mucosa in this study, are involved in the response to pathogens. The activation of M1 macrophages may contribute to the reduction of opportunistic pathogens in the colon mucosa as discussed above, or simply indicate the development of innate immunity in the colon mucosa during the first 5 d of life. Also, “Pathogen Induced Cytokine Storm Signaling Pathway” was predicted to be activated at D5. Cytokine storm is a systemic inflammatory syndrome that can be triggered by several factors, including antigens [67], suggesting that the occurrence of local inflammation due to other antigens, such as pathogenic viruses in the colon mucosa for the first 5 d after birth. Indeed, functional analysis showed the possibility of viral infection during the first 5 d of life, even though calves with diarrhea were not observed in this study.

IFNs, including *IFN-α*, *IFN-β*, and *IFN-λ*, are involved in antiviral response in intestinal tissues [68, 69]. DE genes involved in the “Interferon Signaling” pathway, including *IFI6*, *IFIT1*, *IFIT3*, *ISG15*, and *MX1*, were found to be expressed at higher levels in the circulating transcriptome of feedlot cattle infected with bovine viral diarrhea virus compared to non-infected cattle [70]. These finding suggest that pathogenic viruses may enter the gastrointestinal tract of calves, which may lead to the activation of interferon signaling in the colon mucosa after the first feeding. Activation of “Role of Hypercytokinemia/hyperchemokinemia in the Pathogenesis of Influenza” also supports the possibility of pathogenic viral infection in the colon mucosa. As a lack of IFN signaling prolonged diarrhea disease during Rotavirus infection in neonatal mice [71], IFN signaling may also be related to the occurrence of diarrhea. However, as we discussed above, these activated pathways related to viral infection may be simply results of immune function development after birth. Therefore, further research is necessary to explore the relationship between IFN signaling, prevalence of pathogenic viruses, and occurrence of diarrhea in neonatal and preweaning calves. It should be noted that IFN-α receptor 1 (*IFNAR1*), IFN-α receptor 2 (*IFNAR2*), and IFN-λ receptor 1 (*IFNLR1*) were expressed in the colon mucosa of neonatal calves in this study; however, their ligands, especially *IFN-λ*, which are thought to play a major role in the antiviral response in the intestine [68], were not detected in D0 and D5 calves (Additional file 1: Table S4). It would be interesting to investigate the point at which these genes become detectable, because they may be associated with diarrhea in calves.

As described in our previous study [16], which utilized the same animals as in this study, the presence of viable *Saccharomyces cerevisiae* was only confirmed in feces of SCB calves, while no colonies of viable SCB were detected in the feces collected from CON calves, suggesting that SCB were successfully fed in this study. The SCB dosage applied in this study positively influenced growth performance or gastrointestinal health in our previous studies [17, 72]. Contrary to our hypothesis, SCB supplementation did not prevent opportunistic pathogens colonization nor promote beneficial bacteria colonization in the colon. In addition, the absolute abundance of *Lactobacillus* in the colon mucosa of D5 SCB calves was lower compared to D5 CON calves. Interestingly, our previous study (using the same animals) [16] showed that this difference between CON and SCB was not observed 2 d later (on d 7 after birth with post-mortem sampling). Although we cannot explain the discrepancy between these results, there are studies indicating that calves (> 5 d of life) fed SCB had higher abundances of *Lactobacillus* in the jejunum and feces compared to calves without SCB supplementation [16, 17, 73]. Therefore, SCB may have a positive effect in the long term (i.e., weaning transition) rather than in the short term (i.e., neonatal periods) or have a positive effect on other gastrointestinal sites. Moreover, no DE genes were detectable between the CON and SCB groups. Since significant differences in the number of IgA^+^ plasma cells in the colon mucosa and in severe diarrhea were found at 7 days and 7 weeks of life in our previous study [16, 17], significant differences in mRNA levels of the colon mucosa by SCB supplementation may be observed after D5. Although no DE genes were found between treatments, pathways related to B cells and IgA production were only enriched in uniquely expressed genes in the D5 SCB group (Fig. 4F). In gut-associated lymphoid tissue, activated B cells differentiate into IgA^+^ plasma cells [74]. In our previous study, IgA^+^ plasma cells were localized in the crypts of the colon [16]. SCB supplementation may facilitate the differentiation of B cells into IgA^+^ plasma cells in the crypts of neonatal calves.

## Conclusions

Taken together, the positive effect of a short SCB supplementation was indetectable compared to the impact of the natural growth on mucosa-attached bacteria and host immune function in colon mucosa collected with biopsies during the first 5 d of life. Opportunistic pathogens likely decreased in the colon mucosa due to multiple factors such as colostrum and milk feeding, intestinal environment, and host immune function after birth. Although activation of immune function-related pathways may be the result of host immune function development, this may suggest the presence of other antigens in the intestine during early life. Further studies focusing on the other antigens and host immune function in the colon mucosa are required to better understand intestinal immune function development during the neonatal period.

### Supplementary Information

**Additional file 1: Table S1.** Primer sequences for bacterial targets in biopsy samples; **Table S2.** Relative abundance of bacteria in the colon mucosa of each calf and d at the phylum level; **Table S3.** Relative abundance of bacteria in the colon mucosa of each calf and d at the genus level; **Table S4.** List of raw reads of the expressed gene in each calf; **Table S5.** List of commonly expressed genes among D0 CON, D0 SCB, D5 CON, and D5 SCB; **Table S6.** List of uniquely expressed genes in D0 CON; **Table S7.** List of uniquely expressed genes in D0 SCB; **Table S8.** List of uniquely expressed genes in D5 CON; **Table S9.** List of uniquely expressed genes in D5 SCB; **Table S10.** List of DE genes between D0 and D5; **Table S11.** List of genes in gene modules which correlated with absolute abundance of colon mucosa-attached bacteria.

**Additional file 2: Fig. S1.** Determination of soft-thresholding power in WGCNA; **Fig. S2.** Uniquely expressed genes of D5 SCB calves; **Fig. S3.** The gene modules which correlated with bacteria in the colon mucosa.

## Data Availability

All 16S rRNA amplicon-sequencing and RNA sequencing data were submitted to DDBJ and the DRA database (BioProject Submission ID: PSUB020302, DRA accession: DRA016269, and BioProject ID: PRJDB15827).

## Abbreviations

ACOD1: Aconitate decarboxylase 1
AGT: Angiotensinogen
APOA1: Apolipoprotein A1
APOB: Apolipoprotein B
ASV: Amplicon sequence variants
BW: Body weight
C3: Complement C3
CCL8: C-C motif chemokine ligand 8
CCL20: C-C motif chemokine ligand 20
CCL25: C-C motif chemokine ligand 25
COL2A1: Collagen type II alpha 1 chain
CD40: CD40 molecule
CPM: Counts per million
CSF2RB: Colony stimulating factor 2 receptor subunit beta
CXCL2: Chemokine (C-X-C motif) ligand 2
CXCL6: Chemokine (C-X-C motif) ligand 6
CXCL8: Chemokine (C-X-C motif) ligand 8
DAVID: Database for Annotation, Visualization, and Integrated Discovery
DE: Differential expressed
DUOX2: Dual oxidase 2
*E. coli*: *Escherichia coli*
FDR: False discovery rate
*F. prausnitzii*: *Faecalibacterium prausnitzii*
GBP4: Guanylate binding protein 4
GO: Gene Ontology
GSDME: Gasdermin E
HLA-DMB: Major histocompatibility complex, class II, DM beta
IFNAR1: IFN-α receptor 1
IFNAR2: IFN-α receptor 2
IFNLR1: IFN-λ receptor 1
IFNs: Interferons
IFI6: Interferon alpha inducible protein 6
IFIT1: Interferon induced protein with tetratricopeptide repeats 1
IFIT3: Interferon induced protein with tetratricopeptide repeats 3
IFIH1: Interferon induced with helicase C domain 1
IgA: Immunoglobulin A
IgG: Immunogloblin G
IL1B: Interleukin 1 beta
IL1RN: Interleukin 1 receptor antagonist
IPA: Ingenuity Pathway Analysis
ISG15: Interferon-stimulated gene 15
ISG20: Interferon-stimulated gene 20
KEGG: Kyoto Encyclopedia of Genes and Genomes
LAP: Lingual antimicrobial peptide
LBP: Lipopolysaccharide binding protein
logFC: Log_2_ fold change
MAF: MAF bZIP transcription factor
MR: Milk replacer
MX1: MX dynamin like GTPase 1
NF-κB: Nuclear factor-kappa B
NLRC5: NLR family CARD domain containing 5
NOS2: Nitric oxide synthase 2
PARP14: Poly(ADP-ribose) polymerase family member 14
PARP9: Poly(ADP-ribose) polymerase family member 9
PCA: Principal component analysis
PoCA: Principal coordinate analysis
PSMB8: Proteasome 20 S subunit beta 8
RSAD2: Radical S-adenosyl methionine domain containing 2
SCB: Saccharomyces cerevisiae boulardii
sIgA: Secretory immunogloblin A
TAP1: Transporter 1, ATP binding cassette subfamily B member
WGCNA: Weighted correlation network analysis
ZBP1: Z-DNA binding protein 1
