# Supplementary material for: Bacteria colonization and gene expression related to immune function in colon mucosa is associated with growth in neonatal calves regardless of live yeast supplementation
Source: J Anim Sci Biotechnol. 2024 Jun 5;15:76. doi: 10.1186/s40104-024-01030-7 (PMC11151515; doi:10.1186/s40104-024-01030-7)
Supplement: Supplementary file 2 — Additional file 2: Fig. S1. Determination of soft-thresholding power in WGCNA; Fig. S2. Uniquely expressed genes of D5 SCB calves; Fig. S3. The gene modules which correlated with bacteria in the colon mucosa. [file 40104_2024_1030_MOESM2_ESM.docx]

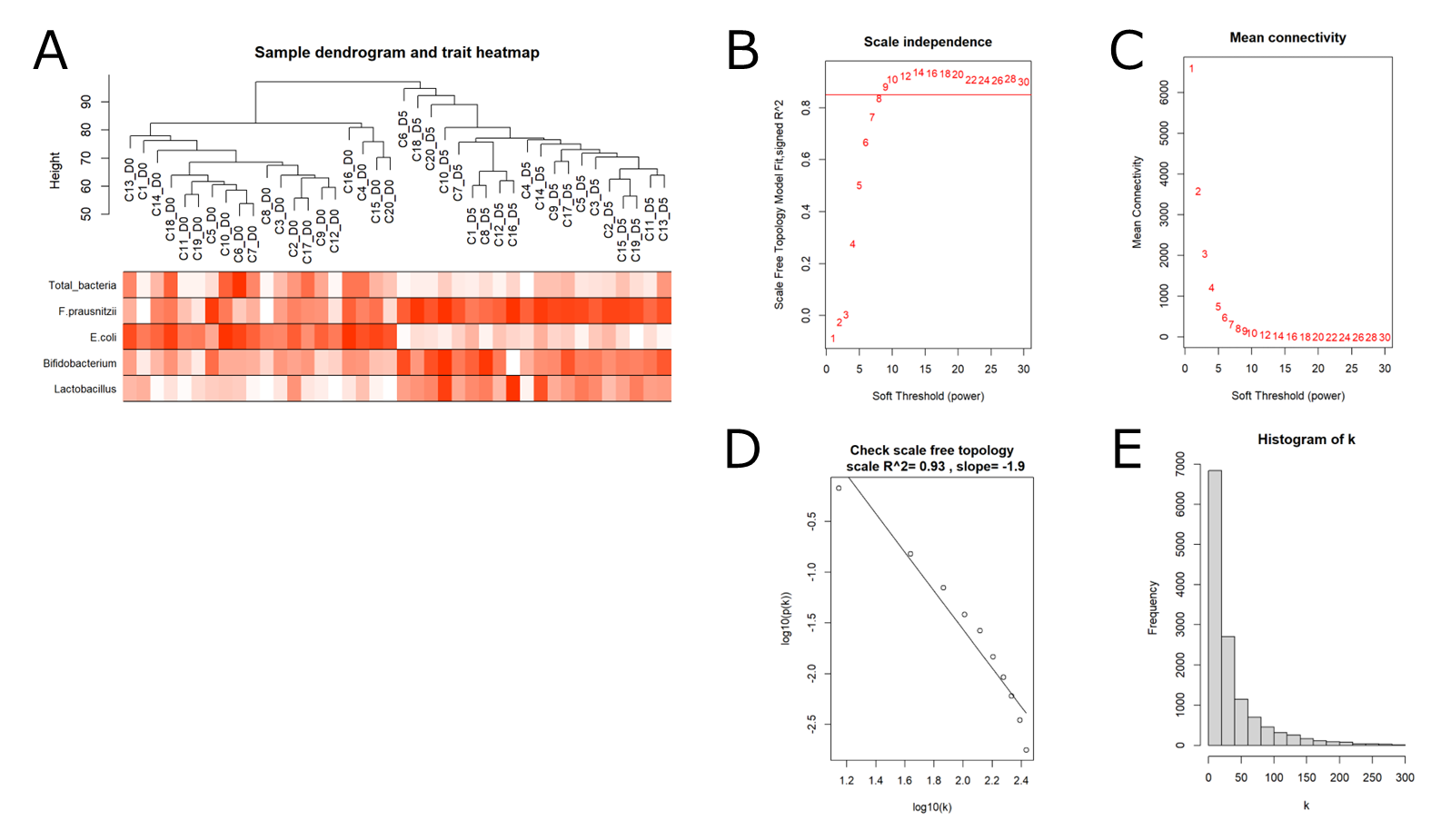
Fig. S1. Determination of soft-thresholding power in WGCNA. (A) Hierarchical clustering dendrogram of colon mucosa transcriptomes (10 D0 CON, 10 D0 SCB, 10 D5 CON, and 10 D5 SCB) and a trait heat map. The gradient from white to dark red in the trait heat map represents low to high bacterial levels. Gray represents missing data. (B) Scale independence, (C) mean connectivity, (D) scale-free topology, and (E) histogram of the connectivity distribution. The R^2^ threshold was set at 0.85 (red line in (B)). WGCNA: weighted correlation network analysis: SCB: *Saccharomyces cerevisiae boulardii*.


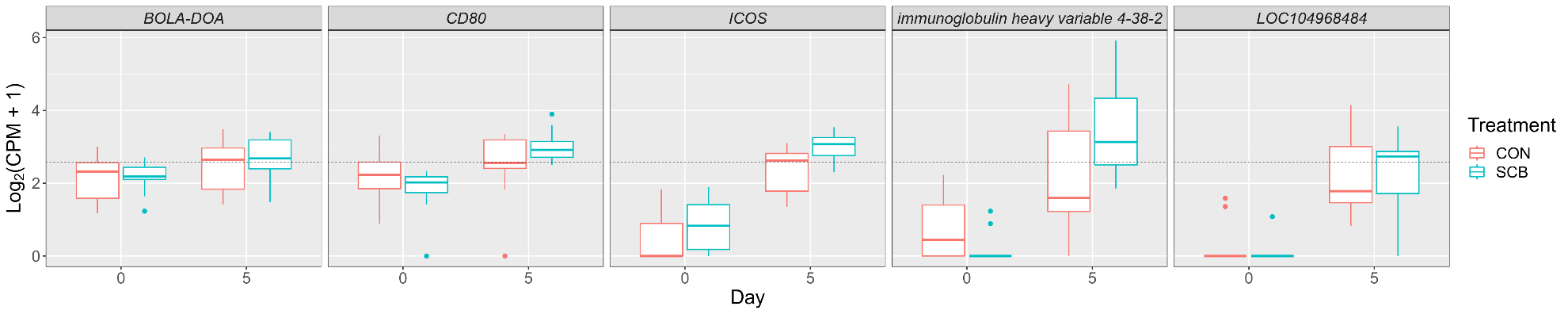
Fig. S2. Uniquely expressed genes of D5 SCB calves. Log_2_(CPM + 1) of genes involved in “Intestinal immune network for IgA production” and “positive regulation of B cell activation,” which were annotated as enriched GO terms and metabolic pathways in uniquely expressed genes of D5 SCB calves. The boxes show the interquartile range between the first and third quartiles and the line inside the box defines the median. Outliers are shown as individual dots. Genes were considered expressed when CPM > 5 (dash-plotted in gray color) in at least half of the animals in each group. SCB: *Saccharomyces cerevisiae boulardii*: CPM: counts per million; IgA: immunoglobulin A; GO: Gene Ontology; *BOLA-DOA*: major histocompatibility complex, class II, DO alpha; *CD80*: CD80 molecule; *ICOS*: inducible T cell costimulator.


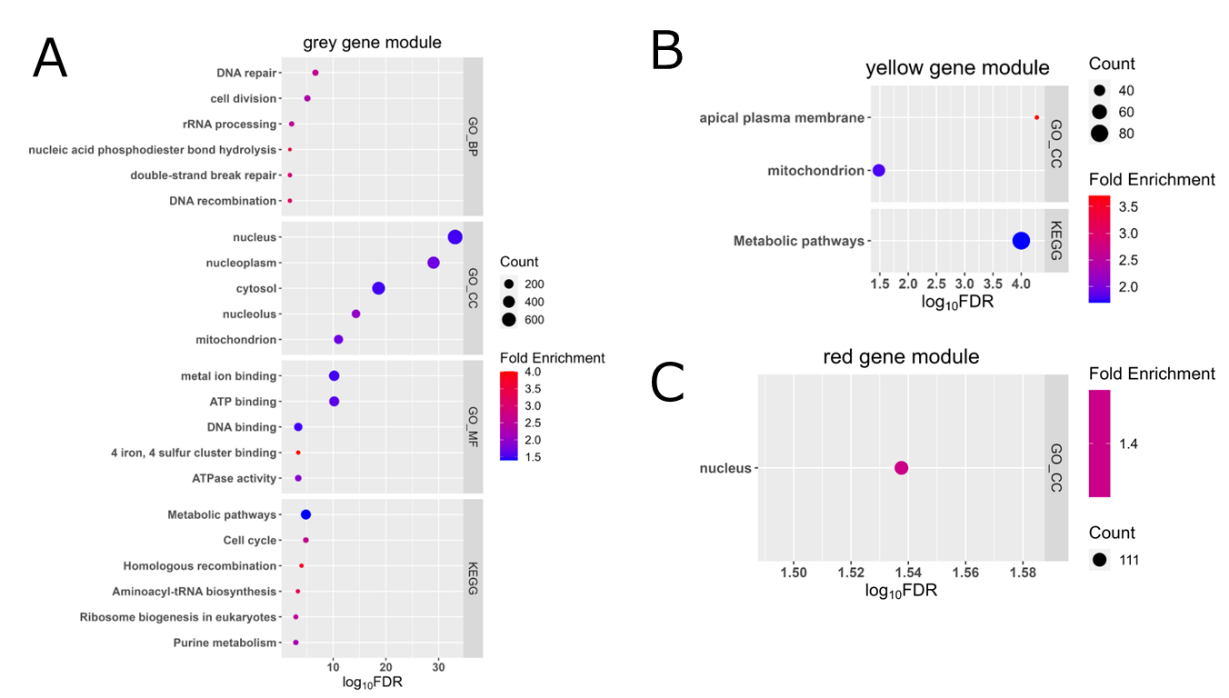
Fig. S3. The gene modules which correlated with bacteria in the colon mucosa (total bacteria, *F. prausnitzii*, *E. coli*, *Bifidobacterium*, and *Lactobacillus*. Enriched cellular components, molecular functions, biological functions, and pathways in the grey (A), yellow (B), and red (C) gene modules, as identified by DAVID. *F. prausnitzii*: *Faecalibacterium prausnitzii*; *E. coli*: *Escherichia coli*; DAVID: Database for Annotation, Visualization, and Integrated Discovery: FDR: false discovery rate.
